# Inhibitory Effects of Columbianadin on Nociceptive Behaviors in a Neuropathic Pain Model, and on Voltage-Gated Calcium Currents in Dorsal Root Ganglion Neurons in Mice

**DOI:** 10.3389/fphar.2019.01522

**Published:** 2020-01-09

**Authors:** Xiaolin Su, Bin Wu, Wentong Zhang, Yong-Hua Ji, Qiuhong Wang, Zhi-Yong Tan

**Affiliations:** ^1^Key Laboratory of Chinese Materia Medica, Heilongjiang University of Chinese Medicine, Harbin, China; ^2^Department of Pharmacology and Toxicology and Stark Neurosciences Research Institute, Indiana University School of Medicine, Indianapolis, IN, United States; ^3^Institute of Special Environment Medicine, Nantong University, Nantong, China; ^4^Lab of Neuropharmacology and Neurotoxicology, Shanghai University, Shanghai, China; ^5^School of Traditional Chinese Medicine, Guangdong Pharmaceutical University, Guangzhou, China

**Keywords:** columbianadin, *Radix angelicae pubescentis*, traditional Chinese medicine, neuropathic pain, calcium currents, oxaliplatin

## Abstract

*Radix angelicae pubescentis* (RAP) has been used in Chinese traditional medicine to treat painful diseases such as rheumatism and headache. A previous study has reported that columbianadin (CBN), a major coumarin in RAP inhibits acute and inflammatory pain behaviors. However, the effects of CBN on neuropathic pain behaviors, and the potential underlying mechanism have not been reported. In the present study, the effects of CBN, compared to another major coumarin of RAP osthole (OST), on oxaliplatin-induced neuropathic pain behaviors and on the voltage-gated calcium currents in small dorsal root ganglion (DRG) neurons were studied in mice. It was found that CBN and OST inhibited both mechanical and cold hypersensitivity induced by oxaliplatin. Moreover, CBN and OST might preferentially inhibit T- and L-type calcium currents (*Ica*). The inhibitory effects of CBN and OST on the oxaliplatin-induced mechanical allodynia were prevented by gabapentin. These results suggest that CBN, as well as OST might inhibit neuropathic pain behaviors through an inhibition of T- and L-type calcium currents in nociceptive DRG neurons.

## Introduction

*Radix angelicae pubescentis* (RAP) is the dried roots of *Angelica pubescens* Maxim. *Freziera biserrata* Shan et Yuan (family Umbelliferae) and was initially described in *Shennong Ben Cao Jing* as a traditional Chinese medicine. RAP has been widely used for the treatments of rheumatism and headache for centuries in China (Chinese Pharmacopoeia Commission, 2015) (Yang et al., 2017). Previous studies found that the extracts of RAP reduce behaviors of acute pain, formalin-induced inflammatory pain, and neuropathic pain in a spared nerve injury model and that coumarins in the extract are the active anti-nociceptive components (Chen et al., 1995; Li et al., 2013; Park et al., 2013; Li et al., 2017). Moreover, a major coumarin in RAP osthole (OST) inhibits pain behaviors in multiple pain models such as inflammatory pain, diabetic pain, and low back pain models (Chen et al., 1995; Wu et al., 2017; Singh et al., 2018; Yuan et al., 2018). These results suggested that OST is an anti-nociceptive component in RAP. On the other hand, columbianadin (CBN) is another major coumarin in RAP and there is one study so far reported that CBN inhibits acute and inflammatory pain behaviors (Chen et al., 1995; Chinese Pharmacopoeia Commission, 2015). However, the effects of CBN on nociceptive behaviors in any neuropathic pain models have not been studied. Moreover, neither the potential mechanism underlying the anti-nociceptive effects of CBN nor if CBN modulates ion channels has been reported.

A variety of ion channels in the nociceptive dorsal root ganglion (DRG) neurons contribute to the transduction, conduction, and transmission of pain signals from periphery to spinal cord. For examples, transient receptor potential (TRP) channels can transduce nociceptive stimuli to electrical signals at free nerve endings that innervate peripheral tissue (Caterina et al., 1997; Patapoutian et al., 2009). Voltage-gated sodium channels are important for the generation and conduction of action potentials from peripheral tissue to spinal cord dorsal horn (Hodgkin and Huxley, 1952; Catterall, 2017). Voltage-gated calcium channels contribute to the release of neurotransmitters and neuromodulators at both central and peripheral termini of DRG neurons (Dolphin, 2016; Gong et al., 2018). Therefore, pharmacological modulators that target ion channels on peripheral sensory neurons, are being pursued as potential candidates for the development of analgesics (Theile and Cummins, 2011; Waxman and Zamponi, 2014). Coumarins have been reported to modulate ion channels. For example, OST exhibits antipruritic effects *via* modulating TRPV_1_ and TRPV_3_ activity (Yang et al., 2016; Sun et al., 2018). Moreover, OST modulates N- and P/Q-type Ca^2+^ channel in the synaptosomes from rat hippocampus (Wang et al., 2008), inhibits L-type calcium current in NG108-15 cell, and inhibits vascular Cav_1.2_ current in smooth muscle cells (Wu et al., 2002; Fusi et al., 2012). On the other hand, another coumarin component of RAP imperatorin inhibits voltage-gated Na^+^ channels in NG108-15 cells (Wu et al., 2013). In contrast to OST and imperatorin, the effects of CBN on any ion channels have not been studied. Moreover, the effects of any coumarins on voltage-gated calcium and sodium currents have not been studied in DRG neurons.

In the current study, we examined the effects of CBN, compared to OST, on mechanical allodynia and thermal hyperalgesia in a neuropathic pain model associated with a chemotherapy drug oxaliplatin. We also studied the effects of CBN and OST on the voltage-gated calcium and sodium currents in DRG neurons.

## Materials and Methods

### Animals

Male C57BL/6 mice at the age of 8 weeks (Jackson Laboratory, Bar Harbor, ME, USA) were used for cell culture and behavior experiments. Mice were housed five or less per cage at a temperature-controlled room (22 ± 0.5°C, 12 h/12 h light/dark cycle) and were with free access to water and pellet diet. All experimental protocols were approved by the Institutional Animal Care and Use Committees of the Indiana University School of Medicine, Indianapolis, Indiana, USA. All procedures were conducted in accordance with the Guide for Care and Use of Laboratory Animals published by the National Institutes of Health and the ethical guidelines established by the International Association for the Study of Pain.

### Von Frey Test

To assess mechanical sensitivity the von Frey assay of “simplified up-down” method was used (Bonin et al., 2014). Mice were placed in Plexiglas cubicle containers on a metal mesh wire platform to allow access to the plantar hind paw. To acclimatize mice to the test procedure, they were placed in the containers for 45 min prior to each test session. A set of eight calibrated von Frey filaments ranging from 0.008 to 6 g (North Coast Medical, Morgan Hill, CA, USA) were applied alternately to the plantar surface of each hind paw until they bent (Li and Sheets, 2018). The duration of each stimulus was approximately 1 s and nociceptive behaviors were considered to be retraction/lifting, rapid shaking, and/or licking of the hind paw.

### Acetone Test

The acetone test was performed according to the method described by previous publications (Kawashiri et al., 2012; Li et al., 2016; Xie et al., 2016). Mice were placed to the same setting described above for the von Frey test and allowed to habituate for 45 min prior to testing. Fifty microliter of acetone was applied to the center of the ventral side of the hind paw and responses were observed. In the first 20 s following acetone application, if the mouse did not withdraw, flick or stamp of the paw then 0 points were recorded for the trial. However, if within this 20 s period the animal responded to acetone, then the animal's response was assessed for an additional 20 s. Responses to acetone were graded according to the following 4-point scale: 0, no response; 1, quick withdrawal, flick or stamp of the paw; 2, prolonged withdrawal or repeated flicking of the paw; 3, repeated flicking of the paw with licking directed at the paw. Acetone test was applied alternately three times to each paw and the responses scored categorically.

### Cell Culture

DRG neurons were dissociated from lumbar L3–L5 ganglia of mice and prepared as previously described (Tan et al., 2006; Qu et al., 2014; Tan et al., 2014). Briefly, mice were sacrificed by exposure to CO_2_ and decapitated. DRG were rapidly removed and placed in bicarbonate free DMEM medium containing digesting enzymes. The DRGs were digested with Liberase TM (0.35 U/ml; Sigma-Aldrich, St Louis, MO, USA) for 20 min before another 15 min with Liberase TL (0.25 U/ml; Sigma-Aldrich, St Louis, MO, USA) and papain (30 U/ml, Worthington Biochemical) at 37°C. After enzymatic digestion, DRGs were triturated using a fire-polished Pasteur pipette. The dispersed cells were resuspended in F12 (Thermo Fisher Scientific, Waltham, MA, USA) medium supplemented with 10% FBS (Thermo Fisher Scientific, Waltham, MA, USA) and 1% penicillin/streptomycin (Mediatech, Inc., Manassas, VA, USA) and plated on polyornithine-laminin-coated coverslips (Discovery Labware, Inc., Bedford, MA, USA). The cells were maintained at 37°C in a humidified 95% air and 5% CO_2_ incubator.

### Electrophysiological Recordings

Patch clamp recordings of small-sized DRG neurons were obtained 14–28 h after dissociation. Cells with a round shape, clean membrane, and without or with minimal processes were chosen. All the neurons were with a diameter less than 25 µm except those used for recording of T-type calcium currents. These neurons were ranging from 20 to 28 µm (**Supplemental Table 2**). Whole-cell voltage clamping was performed as described previously at room temperature (20–22°C) (Tan et al., 2014). Recording electrodes were pulled from borosilicate glass capillaries (Harvard Apparatus, Holliston, MA, USA) using a Sutter P-97 puller (Sutter Instrument, Novato, CA, USA) and the tips were fire-polished to obtain a pipette resistance of 2–5 MΩ when filled with internal solution. A low noise, high-performance Axopatch 200B patch-clamp amplifier (Molecular Devices Corporation, Sunnyvale, CA, USA) driven by a personal computer in conjunction with an A/D and D/A board (DigiData 1320 A series interface, Molecular Devices Corporation) was used to generate and apply voltage pulses to the recorded cells and to record the corresponding membrane currents. To assess the properties of calcium channels, a barium-containing external solution was used to avoid calcium influx-induced rundown of calcium currents. The barium-mediated currents were referred to “calcium currents” throughout the current study. The external solution contained the following components (in mM): 5 BaCl_2_, 140 tetraethylammonium (TEA)-Cl, 2 MgCl_2_, 10 glucose and 10 [4-(2-hydroxyethyl)-1-piperazineethanesulfonic acid] HEPES, pH adjusted to 7.4. The internal solution contained the following components (in mM): 110 Cs gluconate, 30 CsCl, 1 MgCl_2_, and 2 Mg-ATP, 0.1 Na-GTP, 10 HEPES, pH adjusted to 7.2. For sodium current recording, the external solution contained the following components (in mM): 130 NaCl, 30 tetraethylammonium (TEA)-Cl, 3 KCl, 1 MgCl_2_, 1 CaCl_2_, 0.05 CdCl_2_, 10 HEPES, 10 glucose, pH adjusted to 7.4. The internal solution contained the following components (in mM): 140 CsF, 10 NaCl, 1.1 ethylene glycol-bis(β-aminoethyl ether)-N,N,N′,N′-tetraacetic acid, 10 HEPES, pH adjusted to 7.2. The osmolarity of the external solutions and internal solutions were 310–315, and 300–305 mOsm/kg, respectively. All chemicals were obtained from Sigma-Aldrich unless otherwise noted. Calcium currents were evoked by command voltage steps from the holding potential of −100 mV to depolarizing pulses from −80 to +50 mV in 5 mV increments. The duration of pulses is 200 ms and the interpulse time is 3 s. The series resistances of current recording were compensated at 75–80%. Amplitude of calcium currents was measured at the steady-state (averaged value of currents recorded during the last 10 ms of the pulse) for all the conditions except when a mixture of calcium channel blockers (nimodipine, ω-Conotoxin MVIIA, and ω-Agatoxin IVA) was applied together to reveal the transient calcium currents. In the latter condition, the peak amplitude of calcium currents was measured. In the current study, we used a kinetics subtraction method to separate the fast (TTX-S) and slow (TTX-R) components of sodium currents (Cummins and Waxman, 1997). Using this protocol, sodium currents were elicited by 20 ms test pulses to −10 mV after 500 ms prepulses to potentials over the range of −130 to −10. The TTX-R component was obtained at the prepulse of around −50 mV. The TTX-S component was obtained by a subtraction of the TTX-R component from the total current obtained at the prepulse of −110 mV. Although this protocol avoids perfusion of TTX on top of the pre-treatment of coumarins, we recognize the limitation of this protocol that can not separate TTX-S and TTX-R components of sodium currents with 100% accuracy in all the neurons.

### Drugs and Their Administration

**Figure 1:**
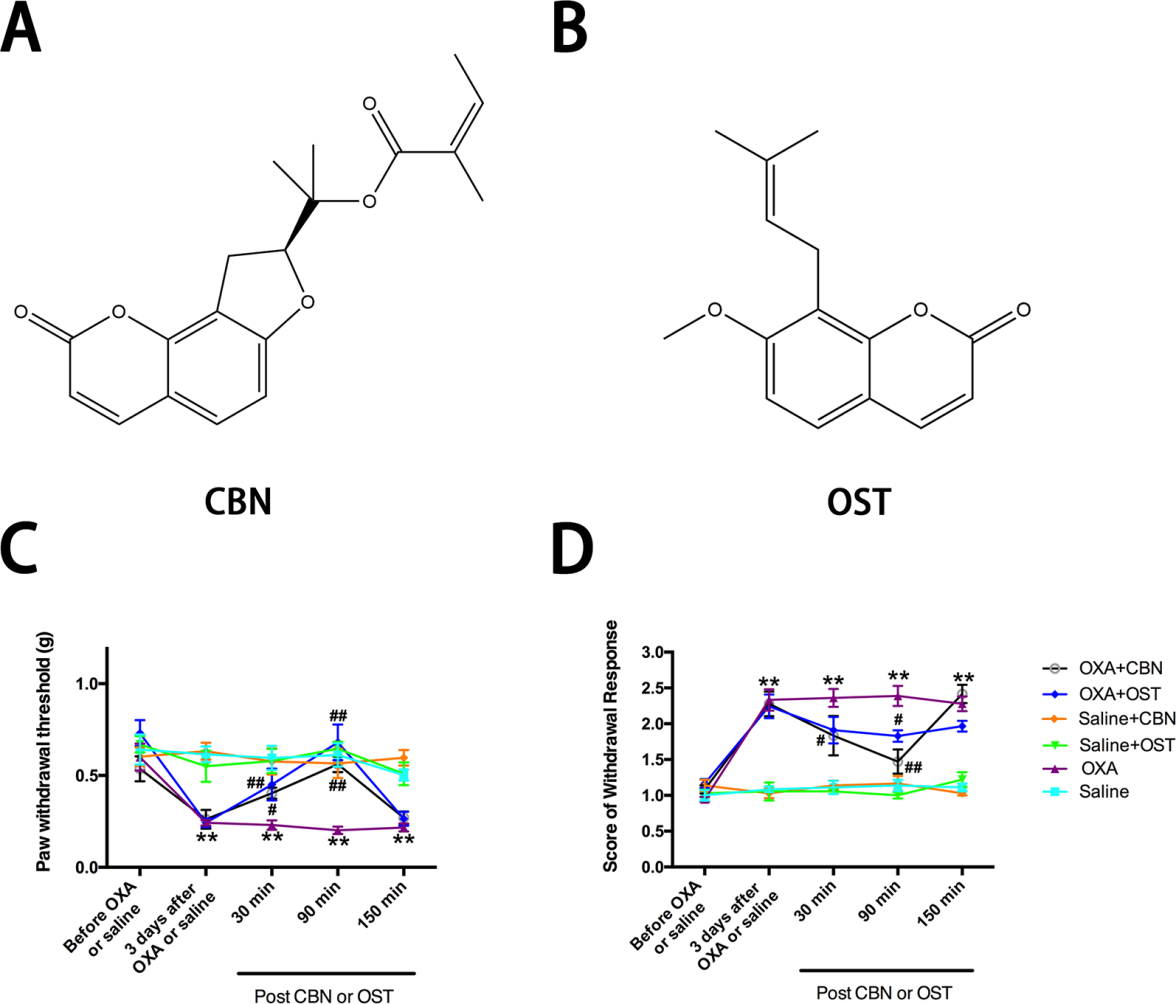
Effects of columbianadin (CBN) and osthole (OST) on oxaliplatin-induced mechanical allodynia and cold hyperalgesia. **(A)** Structure of columbianadin (CBN). **(B)** Structure of osthole (OST). **(C)** Mechanical allodynia, represented by the decrease in paw withdrawal threshold (PWT) upon mechanical stimuli by von Frey filaments, was induced by oxaliplatin (OXA). **(D)** Oxaliplatin-induced cold hyperalgesia (acetone test). The effects of treatments (CBN or OST) on PWT and cold responses were tested 3 days after oxaliplatin or saline (with 5% glucose) administration. Both CBN (10 mg/kg, i.p.) and OST (10 mg/kg, i.p.) significantly reversed oxaliplatin-induced mechanical allodynia and cold hyperalgesia. However, neither CBN nor OST significantly changed mechanical or cold responses in saline-treated animals. Groups of experiments include OXA (oxaliplatin administrated 3 days before the treatment of CBN or OST), saline (saline control for oxaliplatin administrated 3 days before the treatment of CBN or OST), OXA+CBN (CBN administrated 3 days after the treatment of oxaliplatin), OXA+OST (OST administrated 3 days after the treatment of oxaliplatin), saline+CBN (CBN administrated 3 days after the treatment of saline), and saline+OST (OST administrated 3 days after the treatment of saline). Values are represented as mean ± SEM. **p < 0.01, OXA *vs.* saline group; ^##^p < 0.01, *vs.* OXA group. Two-way repeated ANOVA followed by *post hoc* Bonferroni analysis. N = 6 mice per group.

Oxaliplatin, nimodipine, ω-Conotoxin MVIIA, niflumic acid, and gabapentin were purchased from Sigma-Aldrich (St. Louis, MO, USA). ω-Agatoxin IVA were from Tocris, Bio-Techne Corporation (Minneapolis, MN, USA). CBN (purity: 99.85%, Cat. No. HY-N0362) and OST (purity: 99.90%, Cat. No. HY-N0054) were from MedChemExpress (Monmouth Junction, NJ, USA) and the chemical structure of CBN and OST is shown in **Figure 1**. For electrophysiology, the drugs were made in external solution at different dose including CBN (100 µM), OST (100 µM), nimodipine (2 µM), ω-Conotoxin MVIIA (1 µM), and ω-Agatoxin IVA (0.2 µM). To avoid the potential rundown of currents and and/or potential artifact introduced by the decreased quality of voltage clamp over time, all these drugs were pre-treated in the bath solution [contains a final concentration of 0.1% dimethyl sulfoxide (DMSO)] in the recording chamber for 10–60 min before the recording of calcium currents. A 0.1% DMSO bath solution was used as control for these experiments. Recordings of calcium currents are all from independent cells and each cell is only used for a single treatment. For behavior test, stock solutions of oxaliplatin in distilled water (5 mg/ml), were further diluted in 5% glucose to give the final concentration of 1.25 mg/ml. CBN and OST and were dissolved in DMSO and further diluted in saline (to give 2% DMSO) for different final concentrations. A single intraperitoneal (i.p.) injection of oxaliplatin (5 mg/kg) was conducted and the effects of OST and CBN (1–10 mg/kg, i.p.) were examined 3 days after oxaliplatin injection. Gabapentin was administrated (30 mg/kg, p.o., single administration) 3 days after oxaliplatin treatment.

### Statistics

Statistical analysis was performed using GraphPad Prism6 (GraphPad Software, La Jolla, CA, USA). Two-tailed independent Student's t-test, one-way ANOVA with Tukey's multiple comparisons test, or a two-way repeated ANOVA followed by *post hoc* Bonferroni analysis were used. Values of p < 0.05 were considered statistically significant. All data are presented as mean ± SEM.

## Results

### Effects of Columbianadin on Oxaliplatin-Induced Mechanical Allodynia and Cold Hyperalgesia

**Figure 2:**
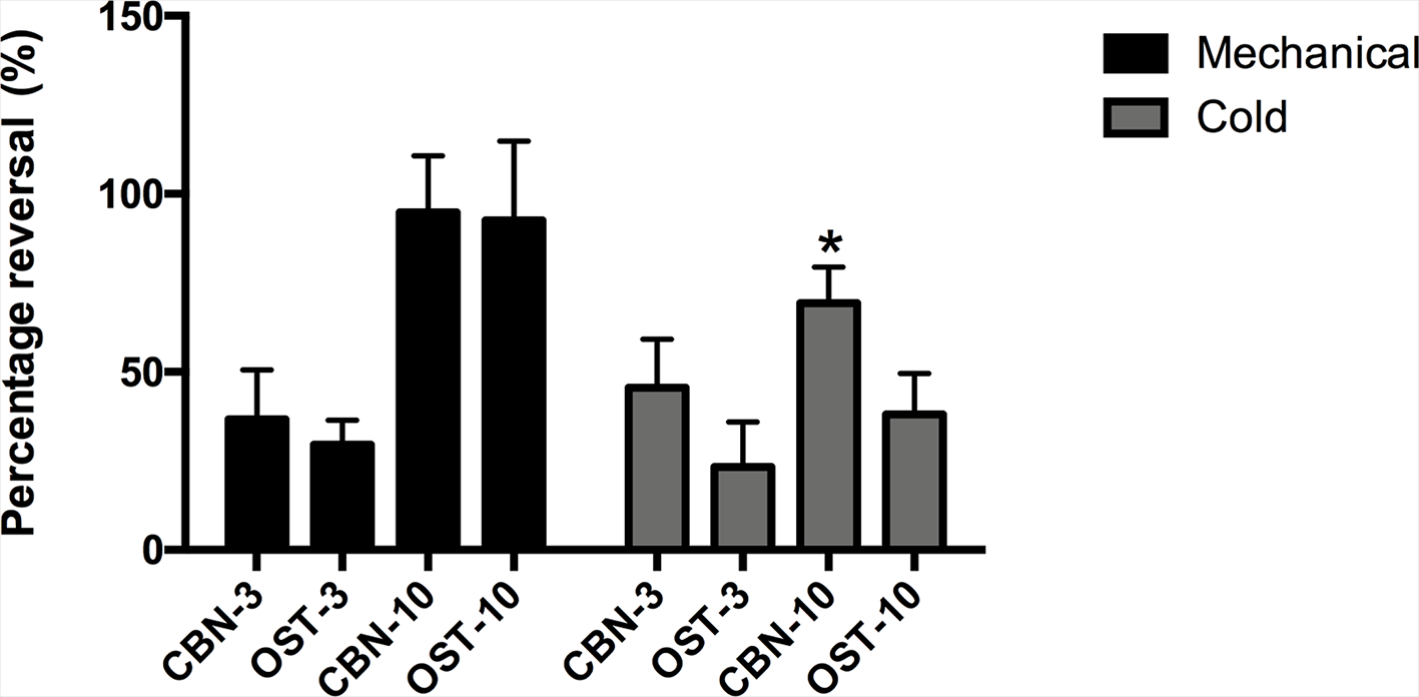
Percentage of the reversing effects of columbianadin (CBN) and osthole (OST) on oxaliplatin-induced mechanical and cold hypersensitivity. The percentage of the reversing effects was calculated by dividing the CBN or OST-induced difference in PWT or cold score by the oxaliplatin-induced difference in PWT or cold score. The effects of CBN and OST at 90 min after injection was used for the calculation of the reversing percentage. CBN-3: 3 mg/kg CBN; CBN-10: 10 mg/kg CBN; OST-3: 3 mg/kg OST; OST-10: 10 mg/kg OST. Values are represented as mean ± SEM. *p < 0.1, *vs.* OST-10, two-tailed independent Student's t-test. N = 6 mice per group.

In the current study, we used an oxaliplatin-induced neuropathic pain model of mouse. We tested the effects of CBN on the mechanical and thermal sensitivity in oxaliplatin-treated mice. We also compared the effects of CBN to OST, the other major coumarin from RAP that has been studied more. As shown in **Figure 1**, oxaliplatin decreased paw withdraw threshold (PWT) to von Frey stimuli and increased score of withdraw response to acetone tested at 3 days post administration. Both CBN and OST significantly reversed oxaliplatin-induced decrease in PWT at 30 and 90 min after administration. The reversing effects occurred at 30 min, reached peak at 90 min, and disappeared at 150 min. At 90 min, both compounds reversed PWT near completely (**Figures 1** and **2**). For the acetone-induced cold responses, a similar time course of reversing effects was observed after treatment of CBN and OST. Significant effects were found at 30 and 90 min after CBN and at 90 min after OST treatment, respectively. The reversing effect of CBN appeared stronger than that of OST at 90 min after treatment (**Figure 2**).

**Figure 3:**
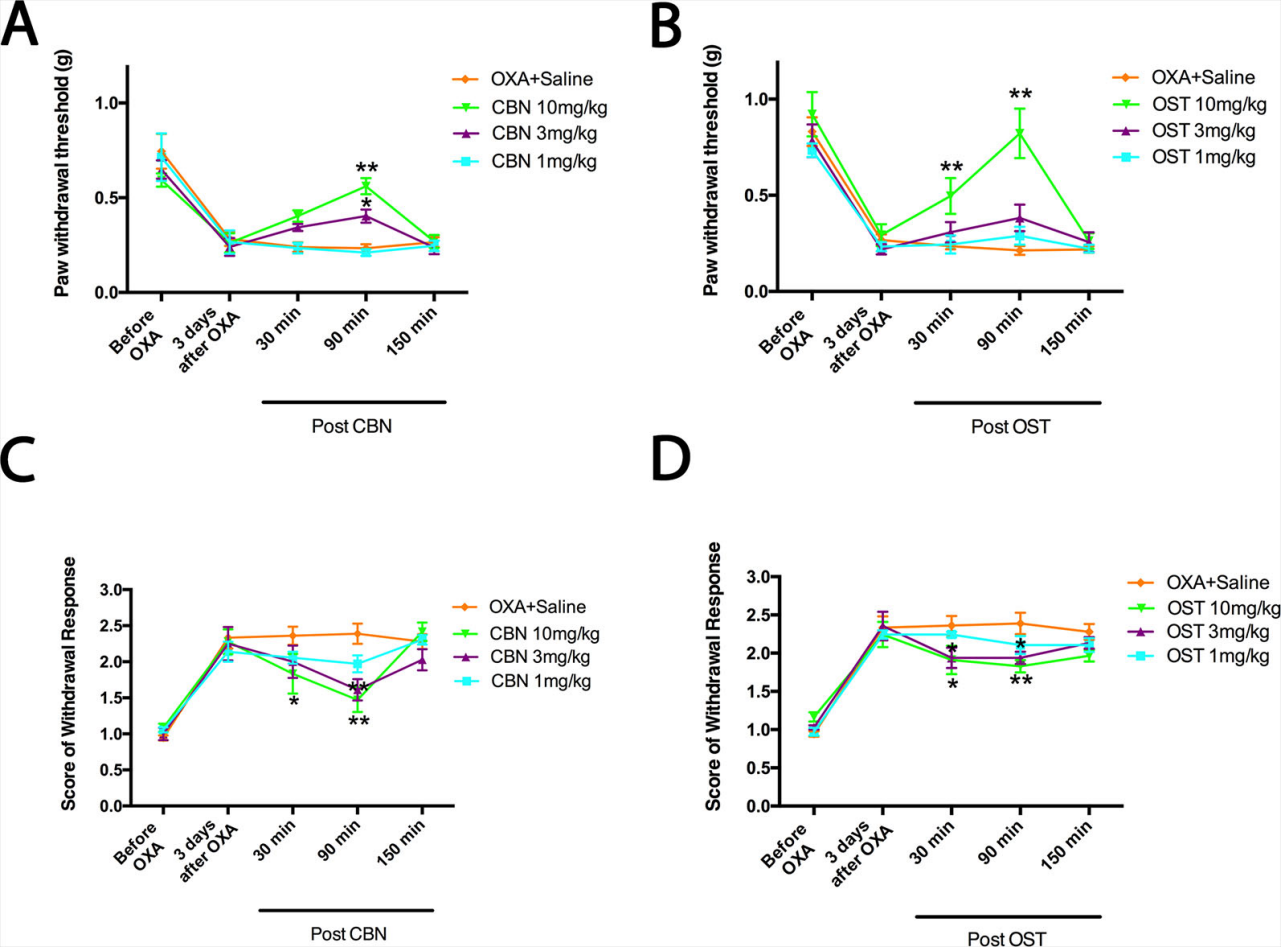
Dose-dependent effects of columbianadin (CBN) and osthole (OST) on oxaliplatin-induced mechanical allodynia and cold hyperalgesia. Effects of treatments (CBN or OST) on pain behaviors were tested 3 days after oxaliplatin administration. CBN and OST dose-dependently attenuated oxaliplatin-induced mechanical and cold pain behaviors. Values are represented as mean± SEM. *p < 0.05, **p < 0.01, *vs.* vehicle, two-way repeated ANOVA followed by *post hoc* Bonferroni analysis. N = 6 mice per group.

We next compared the effects of CBN and OST at different dosages (**Figure 3**). At 3 mg/kg, CBN produced significant effects on both PWT and cold withdrawal score. However, the same dosage of OST only resulted a significant effect on cold response. No significant effects were observed for 1 mg/kg CBN or OST on either pain response. In these behavioral experiments, animals appeared normal other than the inhibition of the sensory hypersensitivity following the treatment of CBN or OST.

### Effects of Columbianadin on *Ica* in Small Dorsal Root Ganglion Cells

**Figure 4:**
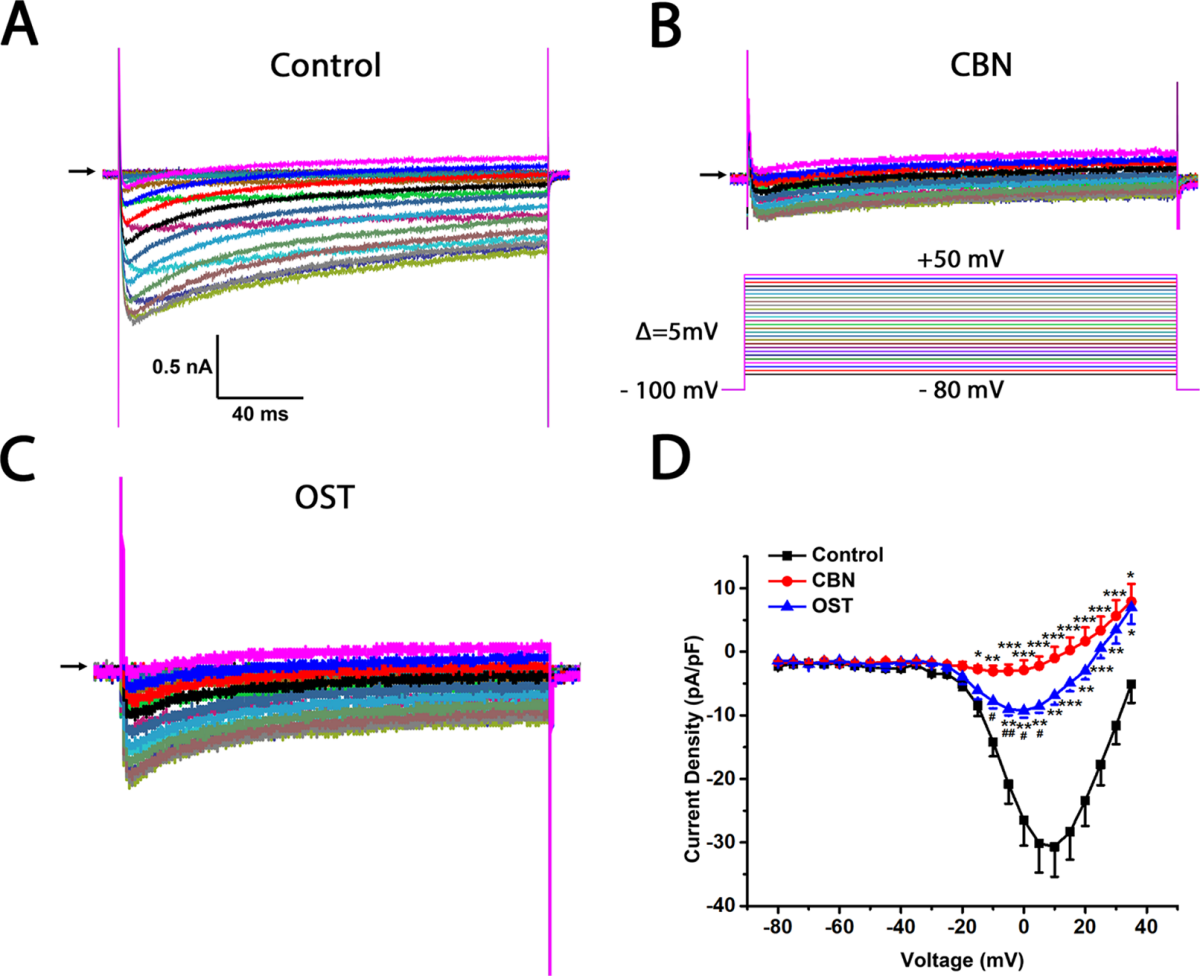
Effects of columbianadin (CBN) or osthole (OST) on the I–V currents of total Ica in the absence of any calcium channel blockers in small dorsal root ganglion (DRG) neurons. **(A)** Representative whole-cell total *Ica* recorded from a small DRG neuron (neuron A) in the absence of any compound. Total *Ica* were obtained by 200-ms depolarizing pulses from a holding potential of −100 mV to test potentials ranging from −80 to +50 mV in a 5 mV increment. The interpulse time is 3 s. **(B**, **C)** Representative *Ica* in the presence of 100 μM CBN or 100 μM OST from two different DRG neurons (neuron B and C, respectively). Recordings are all from independent cells and each cell is only used for a single treatment. CBN or OST were pre-treated for 10–60 min in the recording chamber and maintained during recordings. The time for pre-treating ranged between 10 and 60 min. **(D)** Current-voltage relationships of the total *Ica* in control (black line), OST (blue line), and CBN (red line) groups. Data points are mean ± SEM. *p < 0.05, **p < 0.01, ***p < 0.001 *vs.* control, ^#^p < 0.05, ^##^p < 0.01, *vs.* CBN, two-way repeated ANOVA followed by *post hoc* Bonferroni analysis.

The effects of CBN, compared to OST, on the voltage-gated calcium currents (*Ica*) of small DRG neurons, most of them are presumably nociceptive were studied. The series resistance, cell size, membrane capacitance, cell number, and animal number for whole-cell recording were included in the **Supplemental Tables 1** and **2**. The current-voltage (I–V) relationship of total *Ica* (in the absence of any subtype-specific calcium channel blockers) with or without compounds was firstly compared (**Figure 4**). Compared to control, both CBN and OST significantly suppressed total *Ica*. Moreover, the suppressing effect of CBN were significantly larger than that of OST. The significant voltages of CBN and OST were from −15 to +35 mV, and from −10 to +35 mV, respectively.

**Figure 5:**
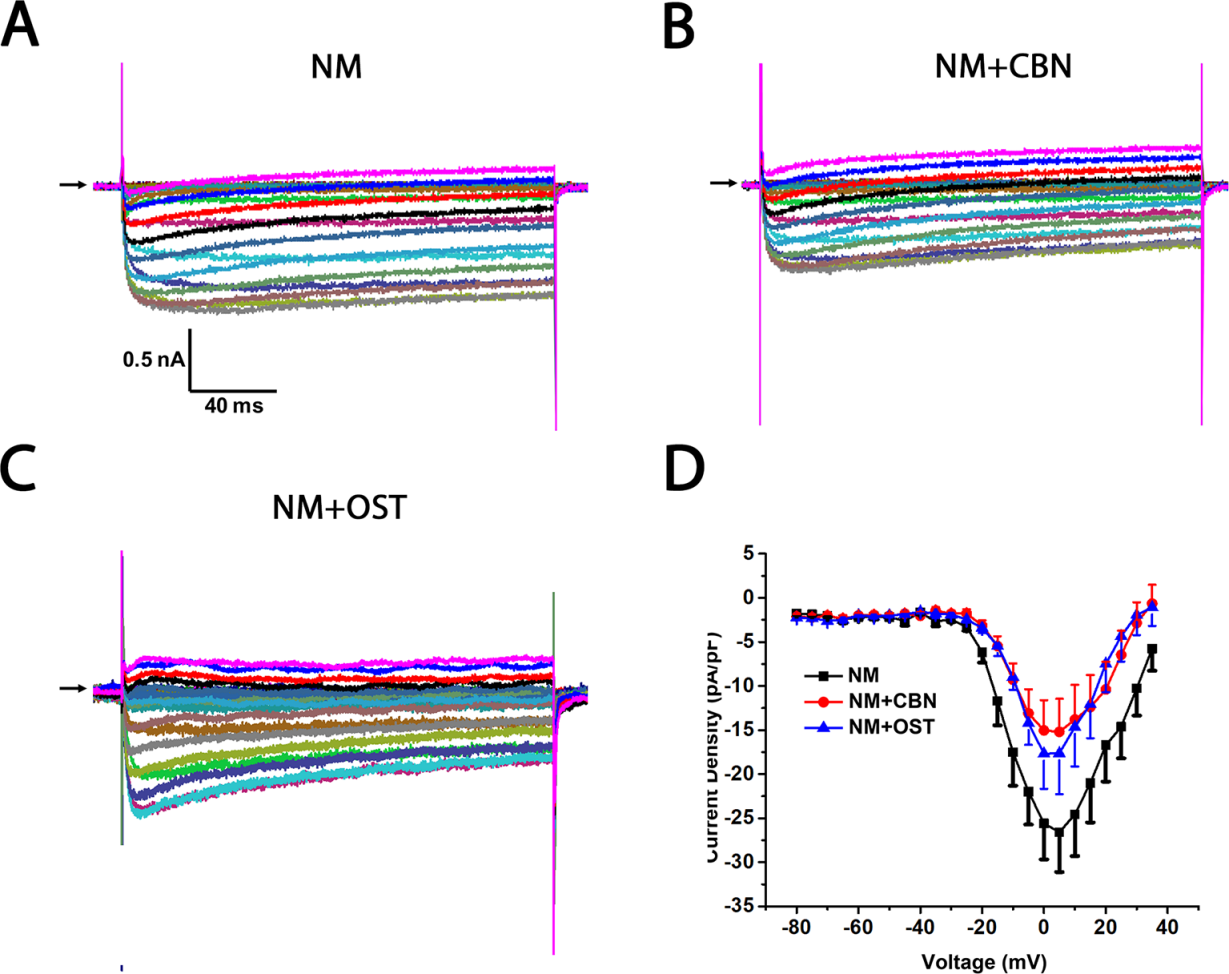
Effect of columbianadin (CBN) or osthole (OST) on the I–V current of Ica in the presence of an L-type Ica blocker nimodipine in small dorsal root ganglion (DRG) neurons. **(A)** Representative whole-cell *Ica* in the presence of 2 μM nimodipine (NM) recorded from a small DRG neuron in the absence of any compound. **(B)** Representative *Ica* in the presence of 2 μM nimodipine and 100 μM CBN. **(C)** Representative *Ica* in the presence of 2 μM nimodipine and 100 μM OST. Recordings are all from independent cells and each cell is only used for a single treatment. Nimodipine, CBN, and OST were pre-treated in the same way described in **Figure 4**. And the same pretreatment method was used for other calcium channel blockers in the following figures. **(D)** Current-voltage relationships of the *Ica* in nimodipine (black line), during exposure 100 μM OST (blue line) and 100 μM CBN (red line). Data points are mean ± SEM. Data are analyzed by two-way repeated ANOVA.

**Figure 6:**
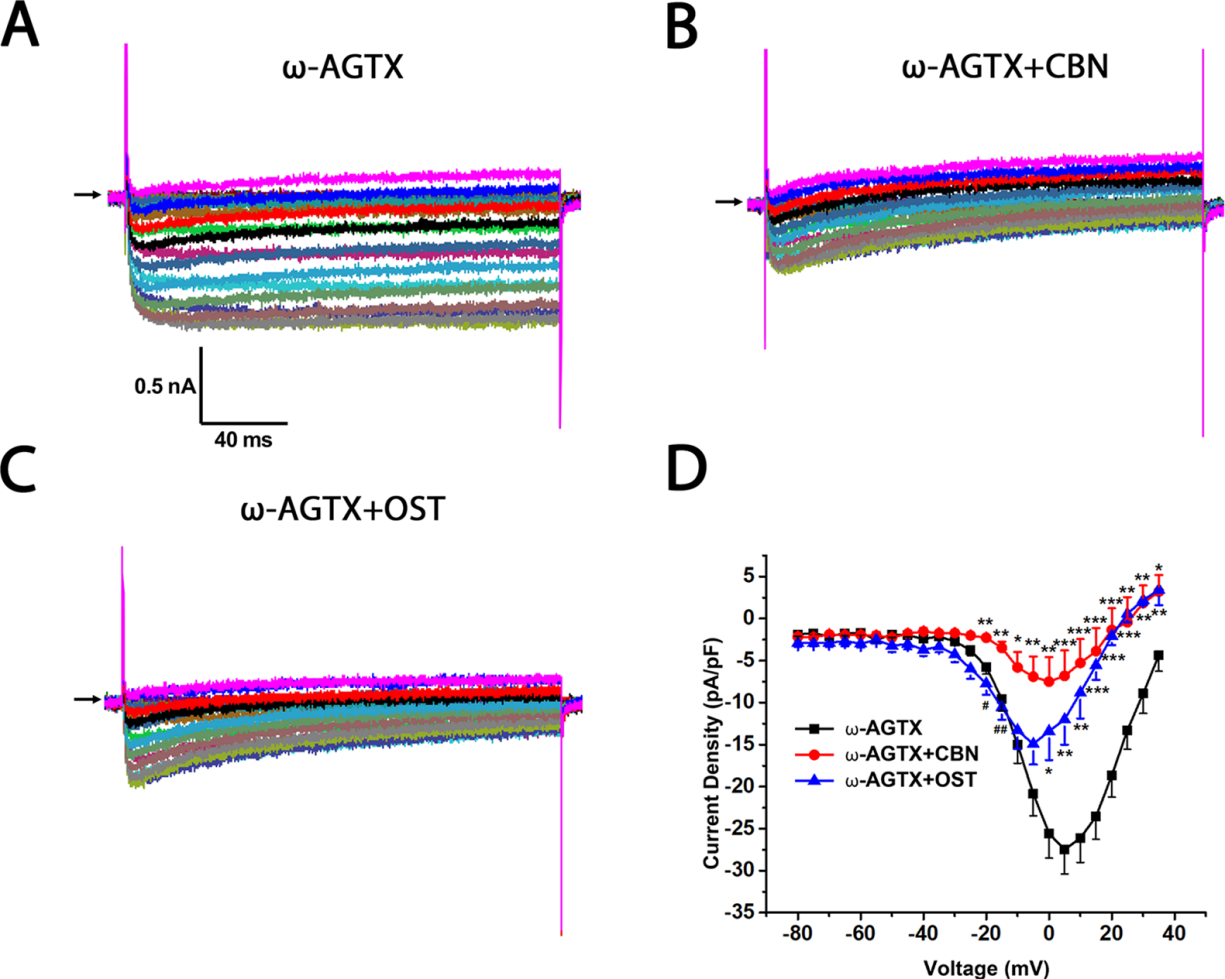
Effect of columbianadin (CBN) or osthole (OST) on the I–V current of Ica in the presence of a P/Q-type Ica blocker ω-Agatoxin IVA in small dorsal root ganglion (DRG) neurons. **(A)** Representative whole-cell *Ica* in the presence of 0.2 μM ω-Agatoxin IVA (ω-AGTX) recorded from a small DRG neuron in the absence of any compound. **(B)** Representative *Ica* in the presence of 0.2 μM ω-Agatoxin IVA and 100 μM CBN. **(C)** Representative *Ica* in the presence of 0.2 μM ω-Agatoxin IVA and 100 μM OST. Recordings are all from independent cells and each cell is only used for a single treatment. **(D)** Current-voltage relationships of the Ica in ω-Agatoxin IVA (black line), during exposure 100 μM OST (blue line) and 100 μM CBN (red line). Data points are mean ± SEM. *p < 0.05, **p < 0.01, ***p < 0.001 *vs.* ω-Agatoxin IVA, ^#^p < 0.05, ^##^p < 0.01, *vs.* ω-Agatoxin IVA +CBN, two-way repeated ANOVA followed by *post hoc* Bonferroni analysis.

**Figure 7:**
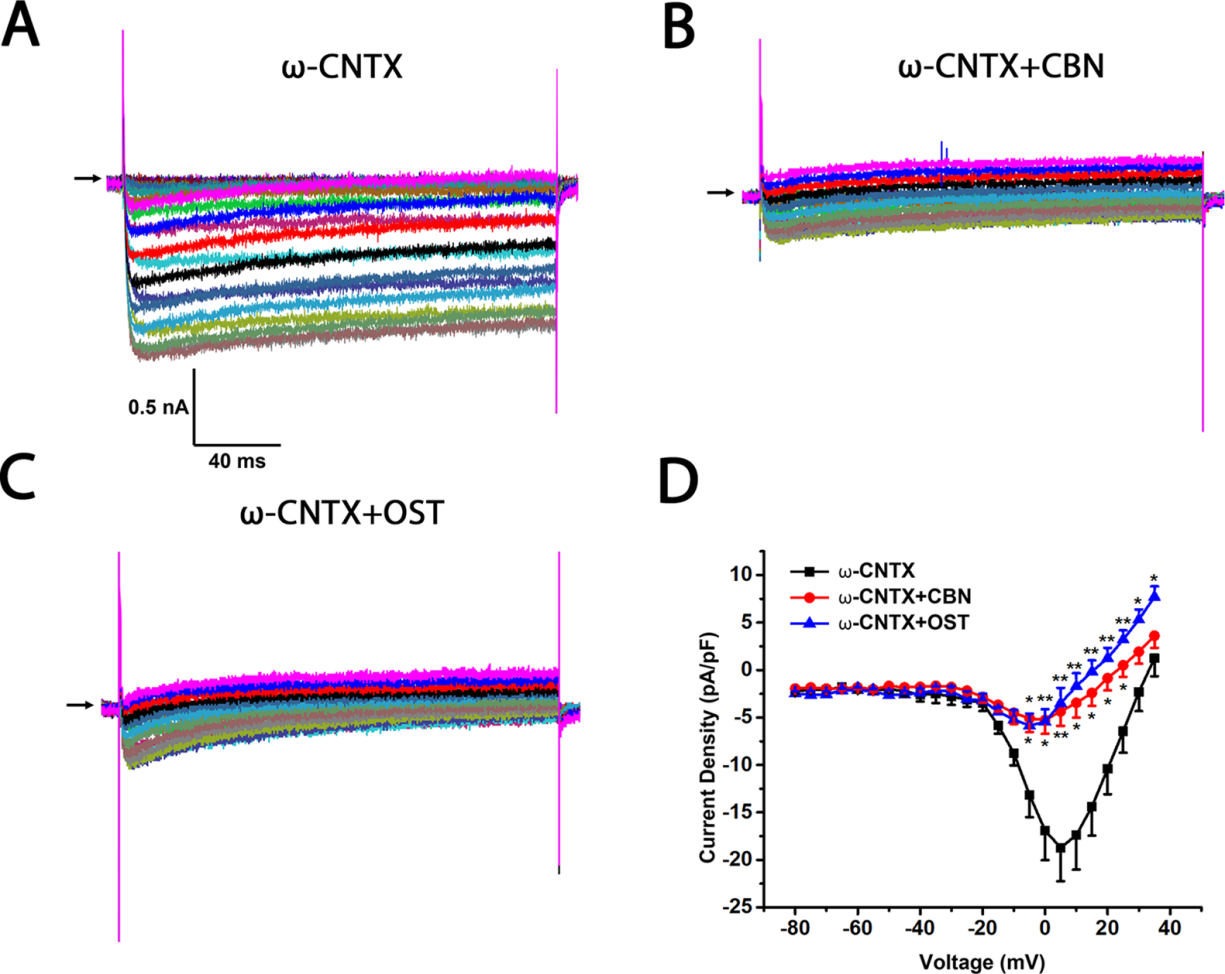
Effect of columbianadin (CBN) or osthole (OST) on the I–V current of Ica in the presence of N-type Ica blocker ω-Conotoxin MVIIA in small dorsal root ganglion (DRG) neurons. **(A)** Representative whole-cell *Ica* in the presence of 1 μM ω-Conotoxin MVIIA (ω-CNTX) recorded from a small DRG neuron in the absence of any compound. **(B)** Representative *Ica* in the presence of 1 μM ω-Conotoxin MVIIA and 100 μM CBN. **(C)** Representative *Ica* in the presence of 1 μM ω-Conotoxin MVIIA and 100 μM OST. Recordings are all from independent cells and each cell is only used for a single treatment. **(D)** Current-voltage relationships of the Ica in ω-Conotoxin MVIIA (black line), during exposure 100 μM OST (blue line) and 100 μM CBN (red line). Data points are mean ± SEM. *p < 0.05, **p < 0.01, *vs.* ω-Conotoxin MVIIA, two-way repeated ANOVA followed by *post hoc* Bonferroni analysis.

**Figure 8:**
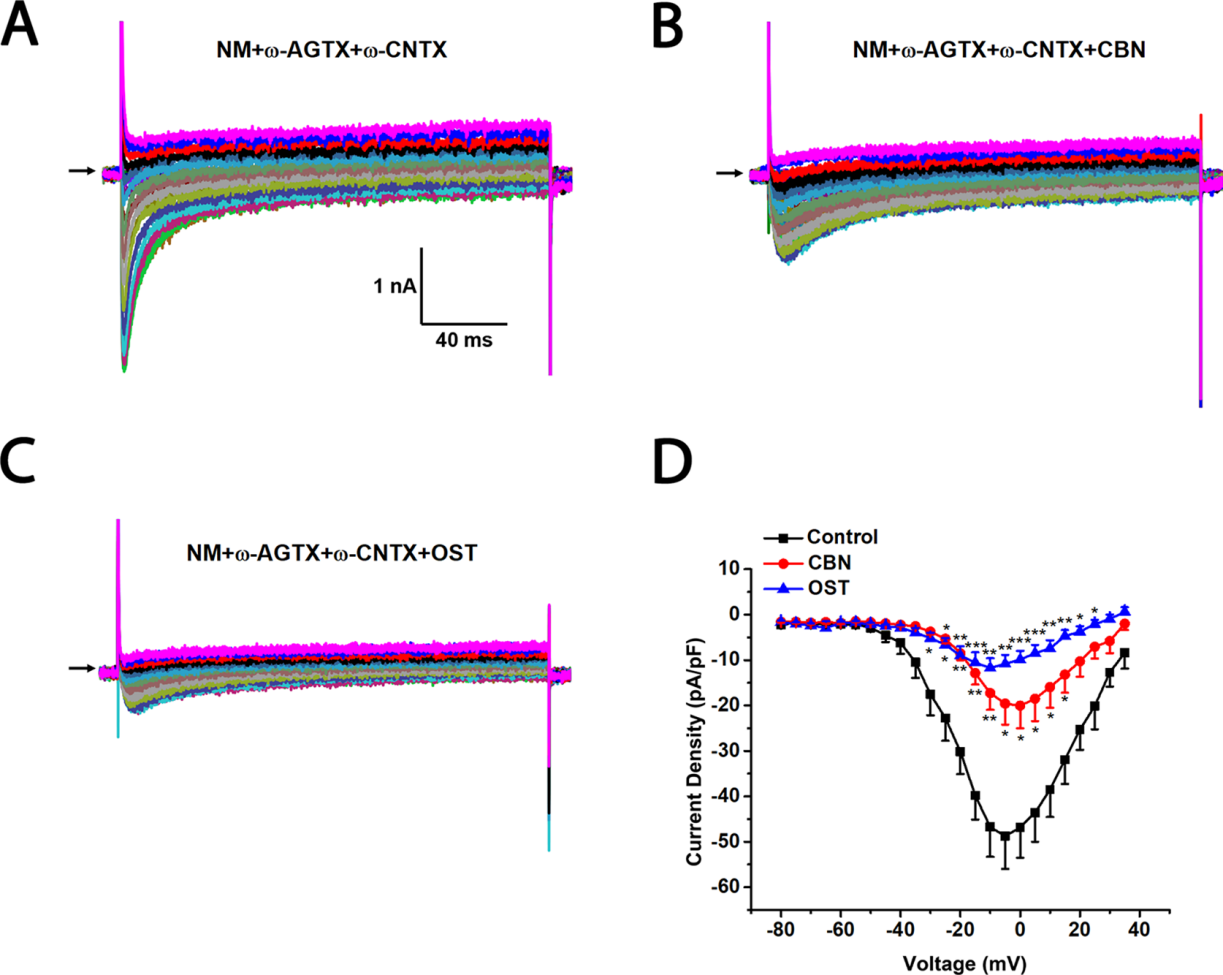
Effects of columbianadin (CBN) or osthole (OST) on the I–V currents of total Ica in the presence of L-, P/Q, and N-type calcium channel blockers in small dorsal root ganglion (DRG) neurons. **(A)** Representative whole-cell total *Ica* recorded from a small DRG neuron in the presence of 2 μM nimodipine, 0.2 μM ω-Agatoxin IVA, and 1 μM ω-Conotoxin MVIIA. **(B**, **C)** Representative *Ica* in the presence of calcium channel blockers and 100 μM CBN or 100 μM OST. Recordings are all from independent cells and each cell is only used for a single treatment. **(D)** Current-voltage relationships of the *Ica* in control (black line), OST (blue line), and CBN (red line) groups. Data points are mean ± SEM. *p < 0.05, **p < 0.01, ***p < 0.001 *vs.* control, two-way repeated ANOVA followed by *post hoc* Bonferroni analysis.

To further study which subtypes of high-threshold calcium currents are suppressed by CBN and OST, we used subtype-specific antagonists of calcium channels. For inhibition of L-type *Ica*, nimodipine was used. For inhibition of P/Q-type *Ica*, ω-Agatoxin IVA was used and for inhibition of N-type *Ica*, ω-Conotoxin MVIIA was used. In the presence of nimodipine, both CBN and OST did not inhibit *Ica* significantly (**Figure 5**). However, a trend inhibition (P < 0.1) was observed at −25 and −20 mV for CBN, and at +25 mV for OST. In the presence of either ω-Agatoxin IVA or ω-Conotoxin MVIIA, both CBN and OST reduced *Ica* significantly (**Figures 6** and **7**). The significant voltages for CBN and OST in the presence of ω-Agatoxin IVA were from −20 to +35 mV, and from 0 to +35 mV, respectively (**Figure 6**). At −20 and −15 mV, CBN and OST showed a significant difference. The significant voltages of CBN and OST in the presence of ω-Conotoxin MVIIA were from −5 to +35 mV, and from −5 to +25 mV, respectively (**Figure 7**). Moreover, in the presence of nimodipine, ω-Agatoxin IVA and ω-Conotoxin MVIIA together, the transient *Ica* was evident (**Figure 8A**). Both CBN and OST reduced the transient *Ica* significantly (**Figure 8**). The significant voltages for CBN and OST to inhibit the transient *Ica* were from −25 to +15 mV, and from −30 to +25 mV, respectively.

**Figure 9:**
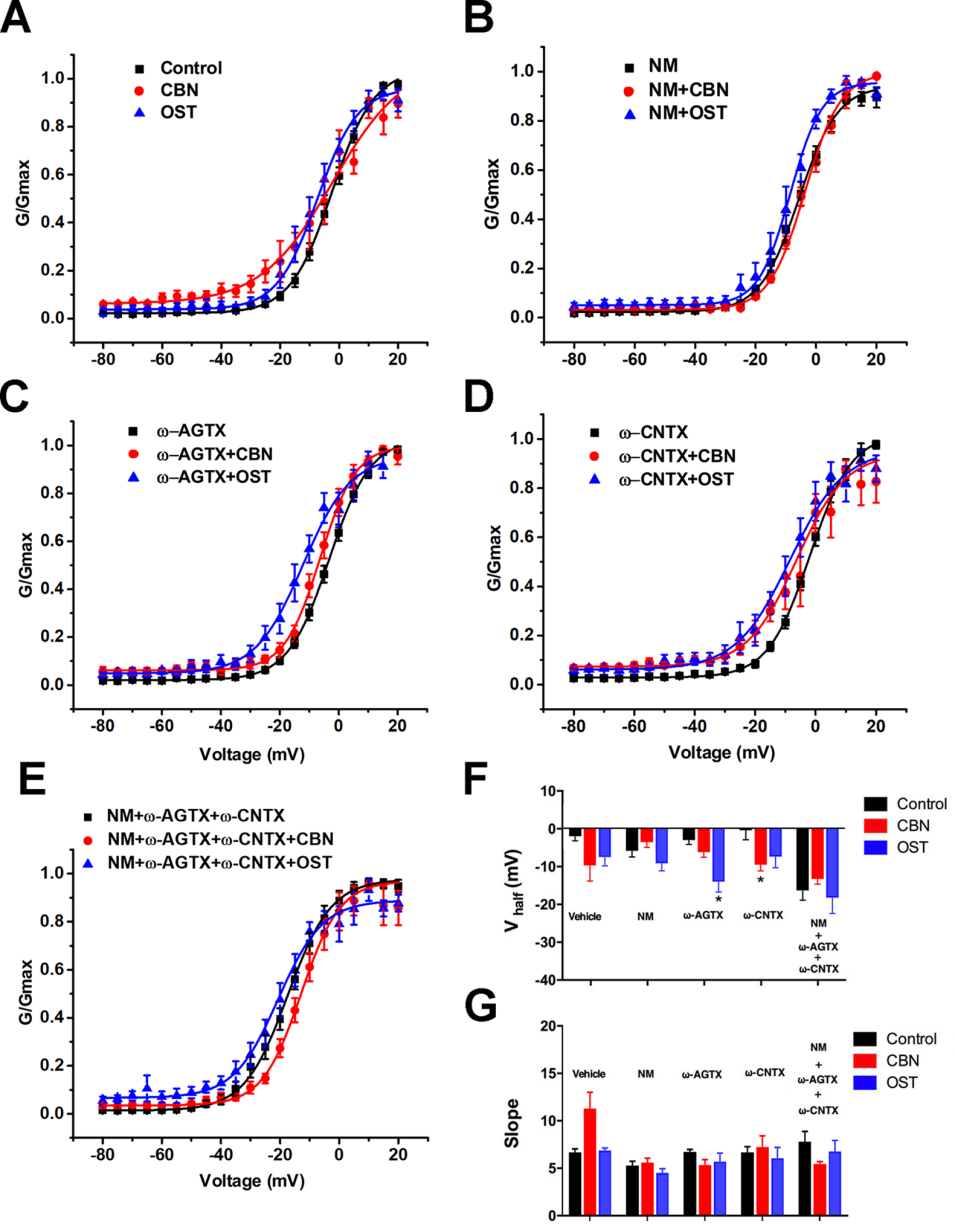
Effect of columbianadin or osthole on the activation of Ica in small dorsal root ganglion neurons. **(A**–**E)** G-V relationships for total *Ica*, *Ica* in the presence of the L-type *Ica* blocker, *Ica* in the presence of the P/Q-type *Ica* blocker, *Ica* in the presence of the N-type *Ica* blocker, and *Ica* in the presence of all types of *Ica* blockers, respectively. Curves from individual cells were fit by a single Boltzmann distribution function. **(F)** Half activation voltages (*V*_half_). **(G)** Slope factors of activation curves. Data were presented as mean ± SEM. *p < 0.05, *vs.* control. One-way ANOVA with Tukey's multiple comparisons test.

In addition to the IV curves, we further compared the activation curves of *Ica* in the absence or presence of calcium channel antagonists (**Figure 9**). The half activation voltage (*V*_1/2_) of the total *Ica*, or the *Ica* in the presence of either nimodipine or the cocktail of blockers, was not changed significantly by either CBN or OST (**Figure 9F**). Moreover, no significant change in *V*_1/2_ was induced by CBN in the presence of ω-Agatoxin IVA or by OST in the presence of ω-Conotoxin MVIIA. However, a hyperpolarizing shift in *V*_1/2_ was observed for CBN in the presence of ω-Conotoxin MVIIA or for OST in the presence of ω-Agatoxin IVA. On the other hand, neither CBN or OST changed the slope factor (*k*) in any conditions significantly (**Figure 9G**).

A shiftment of reversal potential and/or peak voltage of calcium currents was observed in the presence of CBN or OST compared to control (**Figures 4D**, **6D**, and **7D**). To test if these changes might be mediated by chloride currents activated at depolarizing voltages, we used a chloride channel blocker niflumic acid (Hilaire et al., 2005). We found that in the presence of 100 µM niflumic acid, CBN-induced changes in reversal potential and peak voltage of calcium currents were largely reversed (**Supplemental Figure 1**).

### Effects of Columbianadin on Voltage-Gated Sodium Current in Small Dorsal Root Ganglion Cells

**Figure 10:**
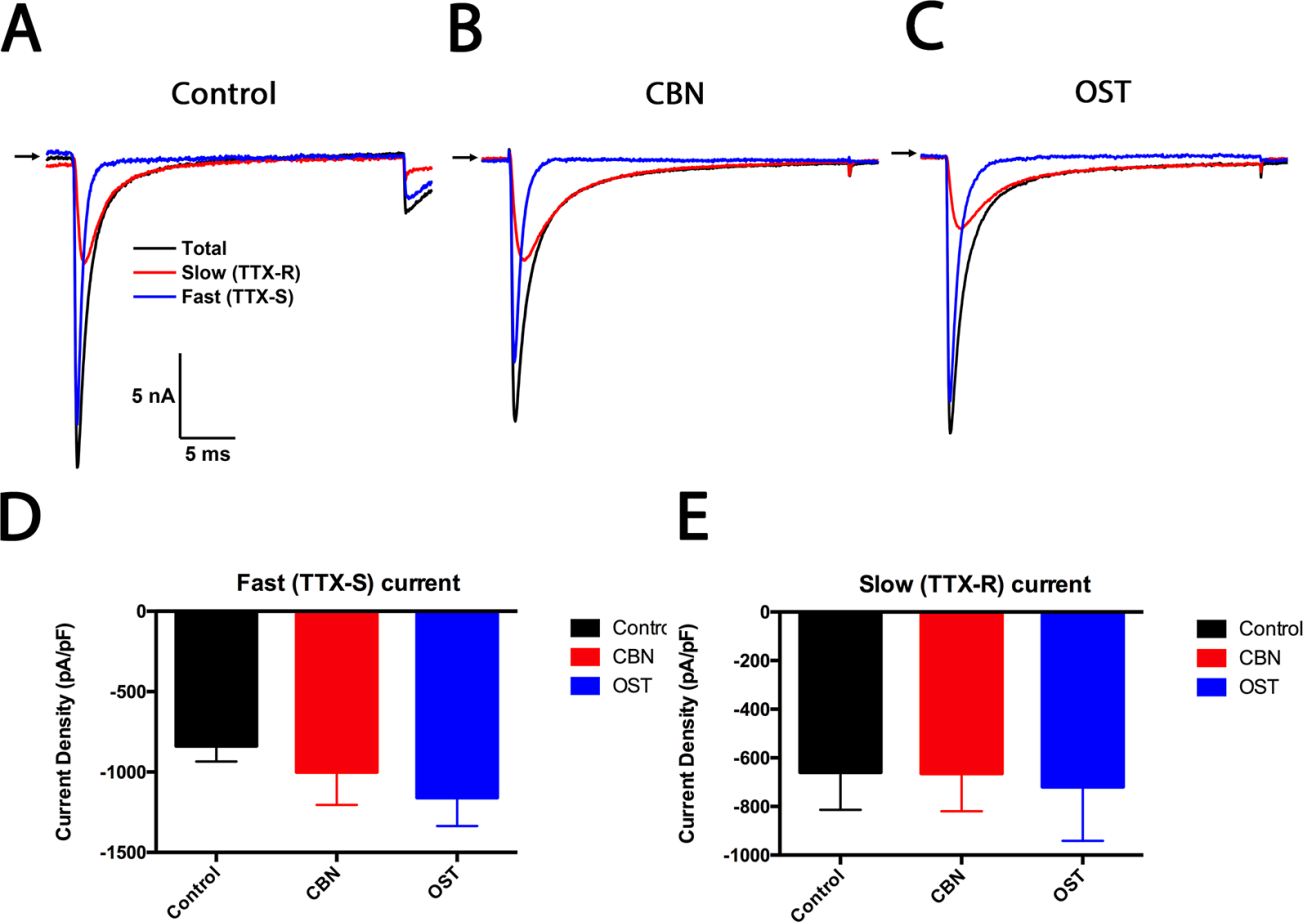
Effects of columbianadin on the fast current (the TTX-S current) and the slow current (the TTX-R current) in small dorsal root ganglion (DRG) neurons. **(A)** Representative total, TTX-S and TTX-R current recorded from a small DRG neuron in the absence of any compound. **(B**–**C)** Representative total, TTX-S and TTX-R current in the presence of 100 μM columbianadin (CBN) or 100 μM osthole (OST), respectively. Recordings are all from independent cells and each cell is only used for a single treatment. **(D**–**E)** Current density of TTX-S and TTX-R current in control, CBN and OST groups. Data were presented as mean ± SEM. One-way ANOVA with Tukey's multiple comparisons test.

To study the effects of CBN and OST on both the tetrodotoxin-sensitive (TTX-S) and tetrodotoxin-resistant (TTX-R) sodium currents, a kinetics subtraction protocol was used (Cummins and Waxman, 1997). As shown in **Figure 10**, the fast current (the TTX-S current) was obtained by subtraction of the slow current (the TTX-R current) from the total current in small DRG neurons. Compared to control, neither CBN nor OST significantly inhibited either the fast TTX-S current or the slow TTX-R current.

### Effects of Columbianadin on Oxaliplatin-Induced Mechanical Allodynia Following Calcium Channel Inhibition

**Figure 11:**
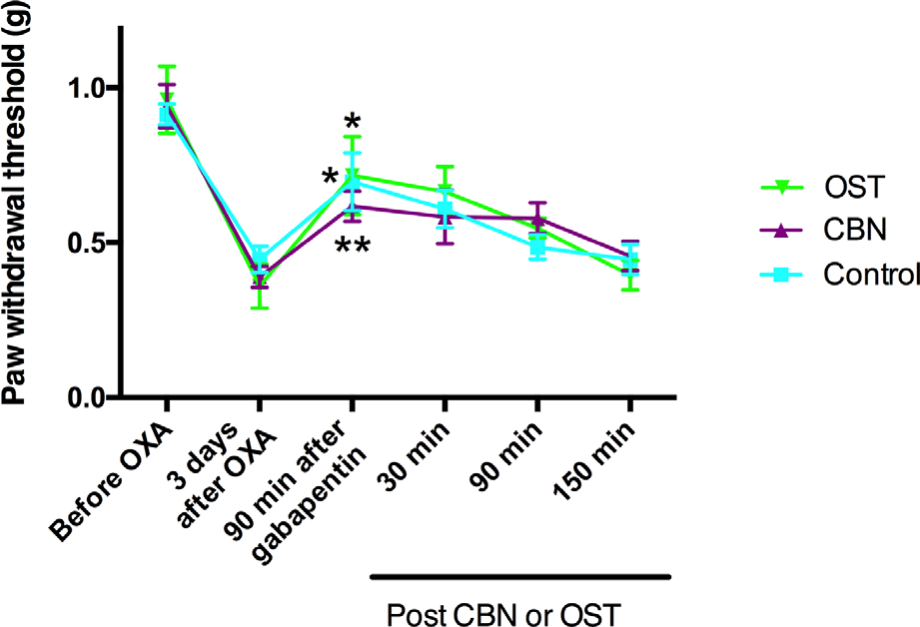
Effects of columbianadin (CBN) and osthole (OST) on oxaliplatin-induced mechanical allodynia in the presence of gabapentin. Mechanical allodynia was induced by oxaliplatin (OXA). Gabapentin was administrated (30 mg/kg, p.o., single administration) 3 days after oxaliplatin treatment. CBN (10 mg/kg), OST (10 mg/kg), or saline (with 2% DMSO) was administrated (i.p.) 90 min after the treatment of gabapentin. Both CBN and OST (10 mg/kg, i.p.) did not change PWT compared to saline group (two-way repeated ANOVA). Values are represented as mean± SEM. *p < 0.05, **p < 0.01, 90 min post-gabapentin *vs.* pre-gabapentin; paired student *t* test. N = 6 mice per group.

Our behavior and cellular electrophysiology experiments suggested that the effects of CBN and OST on neuropathic pain behaviors might be mediated by inhibiting calcium channels. To test this suggestion, we further examined the effects of CBN and OST on the oxaliplatin-induced mechanical allodynia following the treatment of an Food and Drug Administration-approved calcium channel blocker gabapentin. Gabapentin is a first-line treatment for neuropathic pain associated with peripheral neuropathy in clinics while patients with this type of pain often do not respond to nonsteroidal anti-inflammatory drugs or weak opioids. Gabapentin (30 mg/kg, p.o.) significantly inhibited oxaliplatin-induced mechanical allodynia (**Figure 11**). However, neither CBN or OST changed the PWT significantly following the treatment of gabapentin compared to the control (**Figure 11**).

## Discussion

In the present study, we first studied the effects of CBN and OST on the nociceptive behaviors in a mouse model of neuropathic pain induced by oxaliplatin. Oxaliplatin is a platinum-based chemotherapeutic agent commonly used in the treatment of colorectal cancer. However, use of oxaliplatin causes a peripheral neuropathy that is associated with a characteristic sensory hypersensitivity triggered or exacerbated by cold temperature (Argyriou et al., 2013; Boyette-Davis et al., 2018). Like other types of neuropathic pain, current treatments for oxaliplatin-induced pain are often not effective or with serious side effects. In the present study, we found that both CBN and OST significantly inhibited both mechanical and cold hypersensitivity induced by oxaliplatin. Moreover, it appears that the CBN produced a stronger inhibition on cold hyperalgesia compared to OST. These results suggest that CBN is effective for both mechanical and cold sensitivity and OST seems more effective for mechanical sensitivity.

As peripheral sensitization is a common mechanism for neuropathic pain, we aimed to test the potential effects of CBN and OST on ionic currents in DRG neurons. In the previous studies, OST was found to modulate TRPV channels in DRG neurons and to modulate calcium channels in other cells (Wu et al., 2002; Wang et al., 2008; Yang et al., 2016; Sun et al., 2018). In the present study, we found that both CBN and OST significantly inhibited total calcium currents in small DRG neurons. Moreover, the effects of CBN and OST on the neuropathic pain behaviors were prevented by a calcium channel blocker gabapentin. These results suggest that inhibition of voltage-gated calcium currents in nociceptive DRG neurons might contribute to the inhibitory effects of CBN and OST on oxaliplatin-induced neuropathic pain behaviors, as well as on the nociceptive and/or pruritic behaviors observed in other studies (Chen et al., 1995; Yang et al., 2016; Wu et al., 2017; Singh et al., 2018; Sun et al., 2018; Yuan et al., 2018). The stronger inhibition of calcium currents might contribute to the larger effects of CBN on the oxaliplatin-induced cold hyperalgesia compared to OST.

In the recording of calcium currents particularly when the amplitude of currents are small following CBN and OST, a shiftment of reverse potential and/or peak voltage of currents was observed (**Figures 4D**, **6D**, and **7D**). We further found that the changes in reversal potential and peak voltage of calcium currents were largely reversed by a chloride channel blocker niflumic acid (**Supplemental Figure 1**). These results suggest that there might be a chloride current activated at depolarizing voltages that contribute to the changes in the reversal potential and peak voltage of calcium currents especially when the amplitude of steady-state calcium currents is small.

We further tested which subtypes of calcium currents might be involved in the effects of CBN and OST. It has been reported that OST inhibited L-type calcium current in NG108-15 neuronal cells (Wu et al., 2002) and modulated N-type and P/Q type calcium channels in the synaptosomes from rat hippocampus (Wang et al., 2008). In the present study, we found that both CBN and OST significantly inhibited the transient (T-type) calcium currents. For the steady-state currents, CBN and OST might show some preference to L-type, compared to N- and P/Q-type currents since significant inhibition was observed in the presence of either N- or P/Q-, but not L-type blockers. Overall, these results suggest that CBN and OST might preferentially inhibited T- and L-type calcium currents in small DRG neurons.

Calcium channel blockers have been found to inhibit oxaliplatin-induced pain and/or neuropathy in animal models and in patients. For instances, both L-type and non-selective calcium channel blockers reduce sensory neuropathy induced by oxaliplatin-based chemotherapy in patients (Saif et al., 2010; Tatsushima et al., 2013). Nevertheless, lack of pain-reducing effects has also been reported for non-selective blockers gabapentin and pregabalin (de Andrade et al., 2017; Wiffen et al., 2017). In rodent models of oxaliplatin, T-type, L-type, and non-selective blockers reduce mechanical and/or cold hyperalgesia (Ling et al., 2008; Gauchan et al., 2009; Kawashiri et al., 2012; Aoki et al., 2014; Ohsawa et al., 2014; Ruyang et al., 2015; Di Cesare Mannelli et al., 2017; Sekiguchi et al., 2018). Moreover, *in vitro* pre-treatment of oxaliplatin for 24 h selectively up-regulates T- and L-type calcium channels in DRG neurons (Schmitt et al., 2018). These results suggest that T- and L-type calcium channels play critical role in oxaliplatin-induced neuropathic pain. Therefore, the dual T- and L-type blockers CBN and OST might be promising candidate for the development of effective and safe treatment for oxaliplatin-induced neuropathic pain.

Although the current study suggests that CBN and OST inhibit calcium currents in DRG neurons, the underlying mechanisms are not clear. Previous studies have found that OST can modulate intracellular signal molecules such as cyclic adenosine monophosphate (cAMP), cyclic guanosine monophosphate (cGMP), and protein kinase C (PKC) (Ko et al., 1992; Wu et al., 2002; Wang et al., 2008). However, analogues of cAMP and cGMP do not effectively inhibit calcium currents in NG108-15 cells (Wu et al., 2002). In addition, a positive modulation of PKC (Wang et al., 2008) and calcium channels can not account for the inhibitory effects of CBN and OST in the current study. Overall, it seems that CBN and OST might directly modulate the function of calcium channels by stabilizing the inactivation state of the channels (Ko et al., 1992; Wang et al., 2008). On the other hand, it can not be ruled out that the CBN and OST might inhibit calcium channel through an indirect mechanism.

In the current study, we used gabapentin to study the potential cause and effect relationship between the inhibition of calcium channels and the inhibition of pain behaviors of CBN and OST. However, because the pain behaviors were already reduced by gabapentin itself, the effects of CBN and OST on pain behaviors following gabapentin would be underestimated. Therefore, the current experiments can not rule out that CBN and OST might also inhibit pain behaviors through other mechanisms independent of the inhibition of calcium channels. Future studies are needed to identify the mechanisms underlying the effects of CBN and OST on calcium channels in order to development a strategy to selectively target the effects of CBN and OST on calcium channels.

The current study suggests that CBN and OST might display different efficacy inhibiting calcium channels. Although the parent nucleus of CBN and OST is same (as 1,2-benzopyrone), the side chains are different. There are two side chains in OST including a 7-methoxy and 8-isopentenyl. For CBN, there is a furan in the side chain. Moreover, the CBN can be derived from OST through a synthesis of the furan by the 7-methoxy and 8-isopentenyl after a breakdown of the double bonds in the 8-isopentenyl, combined with a substitution of isopentenic acid at the same time. Therefore, the different but derivable side chains might contribute to the different efficacy of CBN and OST inhibiting calcium channels.

In summary, we found that a major coumarin of RAP, CBN displayed strong anti-nociceptive effects in a neuropathic pain model likely through a preferential inhibition of T- and L-type calcium currents in small DRG neurons.

## Data Availability Statement

All datasets generated for this study are included in the article/**Supplementary Material**.

## Ethics Statement

All experimental protocols were approved by the Institutional Animal Care and Use Committees of the Indiana University School of Medicine, Indianapolis, Indiana, USA. All procedures were conducted in accordance with the Guide for Care and Use of Laboratory Animals published by the National Institutes of Health and the ethical guidelines established by the International Association for the Study of Pain.

## Author Contributions

XS, Z-YT, QW, and Y-HJ conceived the idea. XS, BW, and Z-YT designed the experiments. XS, BW, and WZ conducted experiments. XS, BW, and Z-YT analyzed data. XS and Z-YT wrote the manuscript. All authors were involved in the discussion of the project.

## Conflict of Interest

The authors declare that the research was conducted in the absence of any commercial or financial relationships that could be construed as a potential conflict of interest.

## Abbreviations

CBN, columbianadin; DRG, dorsal root ganglion; OST, osthole; PWT, paw withdrawal threshold; RAP, *Radix angelicae pubescentis*; TTX-R, tetrodotoxin-resistant; TTX-S, tetrodotoxin-sensitive; TRP, transient receptor potential.
