# Supplementary material for: Inhibitory Effects of Columbianadin on Nociceptive Behaviors in a Neuropathic Pain Model, and on Voltage-Gated Calcium Currents in Dorsal Root Ganglion Neurons in Mice
Source: Front Pharmacol. 2020 Jan 9;10:1522. doi: 10.3389/fphar.2019.01522 (PMC6970200; doi:10.3389/fphar.2019.01522)
Supplement: Supplementary file 1 [file DataSheet_1.pdf]

Supplemental table 1: The series resistances of whole-cell patch-clamp recording for calcium currents.

Unit (MΩ)	CTRL	NM	AGTX	CNTX	NM+AG+CN
CTRL	5.82±0.67	7.75±0.60	7.05±1.54	6.13±0.70	3.97±0.67
CBN	7.01±0.75	7.09±0.51	7.14±0.71	6.08±0.51	3.48±0.27
OST	7.71±1.17	8.08±0.70	6.16±1.05	5.73±0.58	3.34±0.38

Data are presented as SEM±Mean. No significant differences were observed among each comparisons (control, CBN, and OST) (One way ANOVA).

Supplemental table 2: Passive properties, cell and animal number of whole-cell patch-clamp recording for calcium currents.

		CTRL	NM	AGTX	CNTX	NM+AG+CN
CTRL	Size (µm)	17.50±0.48	17.61±0.45	18.3±0.47	18.59±0.54	25.10±0.66
	Cm (pA)	17.55±1.15	19.45±1.29	18.89±1.40	22.53±2.39	44.70±3.61
	n_cell	11	19	11	9	10
	n_animal	3	4	3	3	3
CBN	Size (µm)	17.92±0.47	17±0.49	17.55±0.43	18.84±0.44	26.20±0.81
	Cm (pA)	19.51±1.45	17.15±0.58	18.45±1.06	21.25±1.78	46.20±5.80
	n_cell	7	11	7	9	10
	n_animal	3	4	3	3	3
OST	Size (µm)	16.71±0.97	18.9±0.52	17.4±0.62	18±0.60	26.20±0.77
	Cm (pA)	18.63±2.32	18.37±2.04	16.57±1.43	20.80±1.02	45.22±4.06
	n_cell	7	10	7	9	9
	n_animal	3	4	3	3	3

Data are presented as SEM±Mean. No significant differences were observed among each comparisons (control, CBN, and OST) (One way ANOVA).
