# Supplementary material for: Inhibitory Effects of Columbianadin on Nociceptive Behaviors in a Neuropathic Pain Model, and on Voltage-Gated Calcium Currents in Dorsal Root Ganglion Neurons in Mice
Source: Front Pharmacol. 2020 Jan 9;10:1522. doi: 10.3389/fphar.2019.01522 (PMC6970200; doi:10.3389/fphar.2019.01522)
Supplement: Supplementary file 2 [file DataSheet_2.pdf]

Figure S1. Effect of niflumic acid on the I-V current of *Ica* in the presence of CBN in small DRG neurons. (A) Representative whole-cell *Ica* in the presence of 100 µM CBN. (B) Representative *Ica* in the presence of 100 µM niflumic acid (NFA) and 100µM CBN. (C) Current-voltage relationships of the *Ica* in the presence of 100µM CBN (black line), and of 100µM NFA and 100µM CBN (red line). Recordings are all from independent cells and each cell is only used for a single treatment. Data points are mean ± SEM (Cell number: n=8 for CBN and n=9 for CBN+NFA; animal number: n=3). There were no significant differences in cell sizes (18.8±0.4 vs. 19.0±0.3µm), membrane capacitances (18.1±1.8 vs. 19.0±1.3pF), or series resistances of voltage clamping (7.3±1.2 vs. 7.6±0.5MΩ) for the CBN and CBN+NFA groups, respectively (Student *t* test).
