# Supplementary figures and images for: Inhibitory Effects of Columbianadin on Nociceptive Behaviors in a Neuropathic Pain Model, and on Voltage-Gated Calcium Currents in Dorsal Root Ganglion Neurons in Mice
Source: Front Pharmacol. 2020 Jan 9;10:1522. doi: 10.3389/fphar.2019.01522 (PMC6970200; doi:10.3389/fphar.2019.01522)

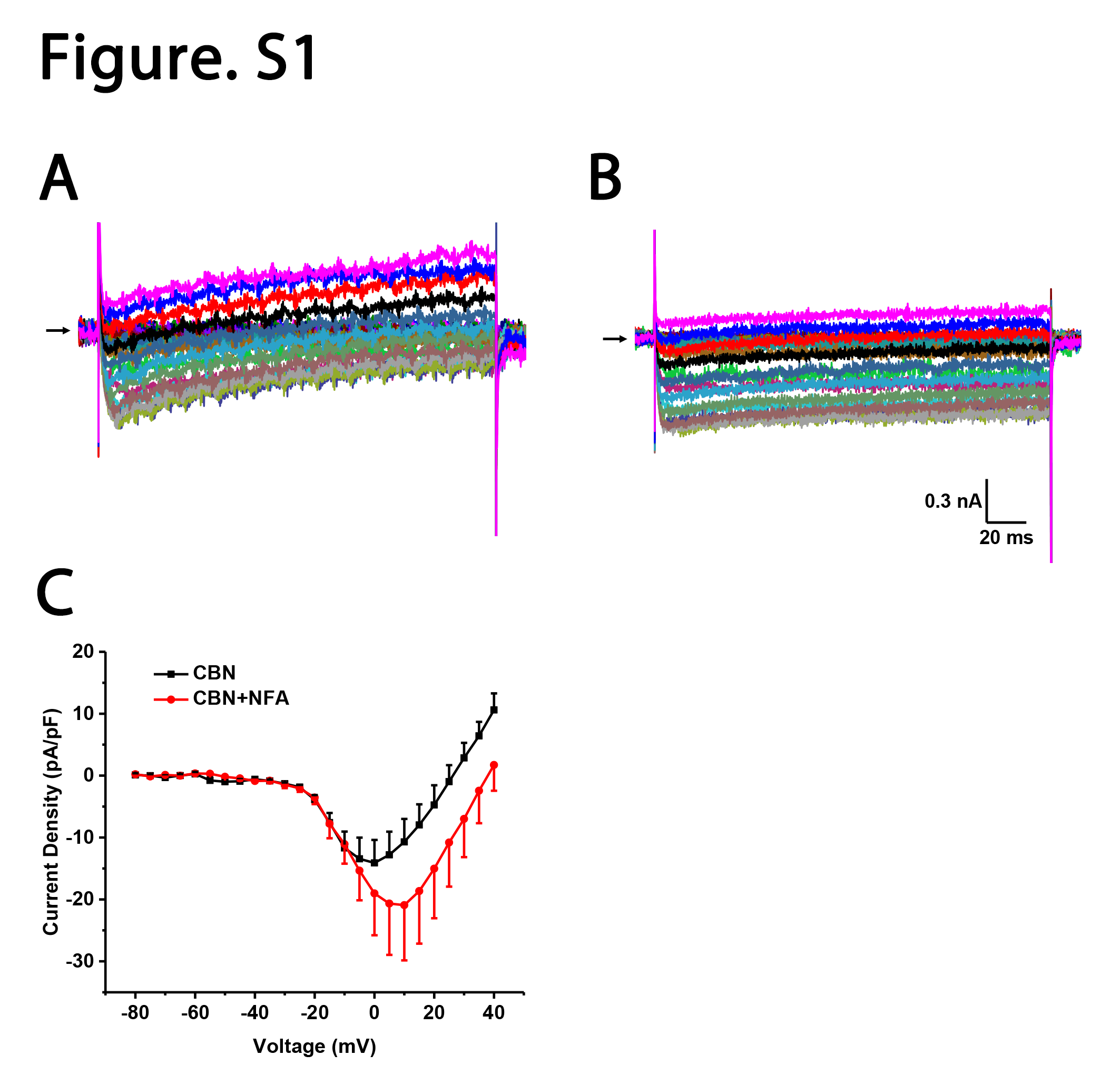

Supplement: Supplementary file 3 [file Image_1.tif]
